# Smartphone-Based Physical Activity Program to Reduce “Chemo-Brain” Symptoms and Improve Health in Cancer Survivors With and Without Type 2 Diabetes: Protocol for a Single-Arm Pre-Post Pilot Trial

**DOI:** 10.2196/79739

**Published:** 2025-12-10

**Authors:** Zachary C Pope, Andriy Yabluchanskiy, Peter Mukli, Michael C Robertson, Jason A Oliver, Sarah J Borengasser, Meghan M Ratke, Jacob E Matney, Chris Mixon, Christina Henson, J Mikhail Kellawan

**Affiliations:** 1 TSET Health Promotion Research Center Stephenson Cancer Center University of Oklahoma Health Campus Oklahoma City, OK United States; 2 Department of Health Promotion Sciences Hudson College of Public Health University of Oklahoma Health Campus Oklahoma City, OK United States; 3 Department of Neurosurgery College of Medicine University of Oklahoma Health Campus Oklahoma City, OK United States; 4 Department of Family and Preventive Medicine College of Medicine University of Oklahoma Health Campus Oklahoma City, OK United States; 5 Department of Pediatrics College of Medicine University of Oklahoma Health Campus Oklahoma City, OK United States; 6 Harold Hamm Diabetes Center University of Oklahoma Health Campus Oklahoma City, OK United States; 7 Department of Health and Exercise Science Dodge Family College of Arts and Sciences University of Oklahoma Norman, OK United States; 8 Department of Radiation Oncology College of Medicine University of Oklahoma Health Campus Oklahoma City, OK United States

**Keywords:** resistance training, aerobic physical activity, wearable technology, smartphone applications, social cognitive theory

## Abstract

**Background:**

The US cancer survivor population is projected to hit 26M by 2040. Chemotherapy is an effective cancer treatment, but can diminish cancer survivors’ quality of life—particularly cognitive function—through select pathophysiological processes, including immune system and antioxidant dysregulation. The resulting cytokine release can impair cerebrovascular function—likely contributing to chemotherapy-induced cognitive impairment (CICI; “chemo-brain”). Type 2 diabetes mellitus (T2DM)—a common cancer survivor comorbidity—shares underlying pathophysiology with CICI. Cancer survivors with T2DM might thus have a higher CICI risk than those without T2DM. Physical activity (PA) counteracts CICI’s and T2DM’s pathophysiology, but little to no research has been conducted assessing the impact of PA on this joint pathophysiology.

**Objective:**

To compare cerebrovascular and cognitive function as well as proinflammatory, cardiometabolic, epigenetic, and psychosocial outcomes between ancer survivors with and without T2DM pre- to postengagement in a 12-week technology-based PA program grounded in the Social Cognitive Theory. We hypothesize that cancer survivors with and without T2DM will demonstrate similar pre to poststudy improvements in psychosocial outcomes, but that changes in cerebrovascular and cardiometabolic outcomes, as well as PA engagement, will be greater for cancer survivors with T2DM. We also believe that each group will have distinct epigenetic profiles that will change pre to poststudy.

**Methods:**

We are conducting a 30-participant pilot study in cancer survivors with (n=15) and without (n=15) T2DM—all of whom report currently experiencing “chemo-brain.” To account for attrition, we are recruiting 38 cancer survivors from Oklahoma City, OK, and the surrounding area. Among the most important eligibility criteria are the self-report of cognitive difficulties following primary cancer treatment, being ≥18 years old, being within 3 years of primary cancer treatment, and not meeting nationally recommended PA guidelines. Participants receive 2 smartphone apps. One smartphone app provides health education and the ability to set goals and journal about their wellness journey. The other provides a workout program continually tailored to each participant via their communication with the study exercise physiologist, with resistance bands and a wearable device provided to support the program. At Baseline and Poststudy, we assess cerebrovascular function (transcranial doppler [TCD]), cognition (National Institutes of Health Toolbox), cardiometabolic outcomes (venipuncture), and epigenetics (saliva collection). Participants also wear accelerometers at Baseline and Poststudy to objectively assess PA, with Baseline, Midpoint, and Poststudy surveys assessing psychosocial outcomes. We will use *t* tests and chi-square tests to assess baseline differences and repeated-measures ANCOVA to assess changes over time.

**Results:**

Participant recruitment started in March 2025, and we expect to recruit until late 2026. We will begin analyzing baseline data in 2026.

**Conclusions:**

Successful study completion will provide valuable insights into the remote delivery of PA-oriented supportive care for cancer survivors experiencing chemo-brain, as well as how T2DM and PA contribute to the mechanistic underpinnings of chemo-brain.

**Trial Registration:**

ClinicalTrials.gov NCT06725953; https://clinicaltrials.gov/study/NCT06725953

**International Registered Report Identifier (IRRID):**

DERR1-10.2196/79739

## Introduction

### Background

While incident cancer cases of any site remain stable, 5-year cancer survival rates have increased [1]. In January 2022, >18M individuals in the US were cancer survivors—defined here as those diagnosed with cancer onward [2]—with a projected increase to 26M by 2040 [3]. Although modern cancer treatments are effective, these treatments can worsen cancer survivors’ health-related quality of life (HRQoL) [4-6]. Several cancer treatments—most notably, chemotherapy—are associated with cognitive difficulties [7-9]. While chemotherapy effectively treats many malignancies, it also negatively impacts vascular endothelial cells [10-12]—leading to pathophysiological processes contributing to cancer survivors’ often-reported chemotherapy-induced cognitive impairment (CICI; aka “chemo-brain”) during/after treatment [7,8]. As the number of cancer survivors increases, CICI mitigation research is crucial to improve HRQoL.

CICI likely occurs for several reasons. Chemotherapy can induce immune and antioxidant dysregulation [13-16]. This dysregulation can lead to peripheral proinflammatory cytokines being released, which damage and cross the blood-brain barrier [13,14,17]—contributing to central proinflammatory cytokine release [18-21] and causing neuroinflammation-related impairments in the myelination process [22]. Endothelial dysfunction and cerebral autoregulation failure can also result from this damage [12,23-25]. Reviews [10,11] suggest these mechanisms alter cerebrovascular function more broadly by impairing cerebral perfusion, glucose metabolism, and angiogenesis; thus contributing to CICI.

Type 2 diabetes mellitus (T2DM) is both a risk factor for developing several common cancers and, given its prevalence, a common cancer survivor comorbidity [26,27], heightening cancer recurrence risk [28,29]. Several pathophysiological mechanisms underlying CICI are observed with T2DM [30]. Insulin resistance can result in repeated hyperglycemic episodes and subsequent proinflammatory cytokine elevation [31-35]—possibly explaining why insulin resistance is correlated with diminished cerebrovascular and cognitive function in individuals with T2DM but without cancer [36-38]. This might also explain why, in newly diagnosed cancer survivors with proinflammatory comorbidities (eg, T2DM), higher proinflammatory cytokines and poorer cognitive function have been observed relative to cancer survivors without comorbidities—even prior to chemotherapy [39]. CICI mitigation research that compares those with and those without T2DM may help clarify the magnitude to which T2DM contributes to the mechanisms underlying CICI. Notably, CICI mitigation research may also benefit from the inclusion of assessments of the epigenome, given that upregulated proinflammatory cytokines, a hallmark of chemotherapy and T2DM, may lead to changes in DNA methylation via histone modification [21,40,41].

Aerobic physical activity (PA) and resistance training (RT) have robust health benefits counteracting much of the pathophysiology underlying CICI and T2DM [42,43]. Reviews [44,45] suggest better cerebrovascular function in those who are more active or fitter relative to those who are less active or fit, with greater fitness positively associated with cognitive function in other at-risk populations [46,47]. In those with T2DM, research has shown that PA is impactful at lowering proinflammatory cytokines [42,48-50] and improving insulin sensitivity [51,52]. Yet, research is needed on how increasing PA in cancer survivors with T2DM reporting CICI improves cerebrovascular and cognitive health through improved fitness. Notably, cancer survivors prefer supportive, individualized, home-based PA programming that includes RT and aerobic activities such as walking [53]. Home-based programs are recommended by the American Cancer Society [54] given their ability to circumvent some of the barriers to PA program participation (eg, distance to clinic, lack of time) among those living with and beyond cancer. Smartphone app technology can assist in providing this home-based PA program access. Relative to routine care, smartphone app–based supportive care among cancer survivors has been observed to improve HRQoL, self-efficacy, and symptoms of distress and depression [55]. Further, multicomponent interventions that include personalized PA programming delivered via smartphone apps, with wearable devices to supplement behavior change, have shown promise for increasing PA among cancer survivors [56,57].

We are conducting a 30-participant pilot study in cancer survivors with T2DM (n=15) and without T2DM (n=15)—all self-reporting “chemo-brain”—that includes personalized PA programming and education delivered via 2 smartphone apps, with a wearable device provided for further behavior change support. We have detailed our Specific Aims and Hypotheses below.

### Specific Aim 1

Characterize cerebrovascular function, cognitive function, proinflammatory cytokines, and cardiometabolic outcome differences between cancer survivors with and without T2DM. We hypothesize that cancer survivors with T2DM will have (1) poorer measures of middle cerebral artery velocity and cerebrovascular conductance, reactivity, and resistance; (2) lower executive function scores; (3) higher C-reactive protein, interleukin-6, and monocyte chemoattractant protein 1 concentrations; and (4) higher blood pressure and insulin resistance.

### Specific Aim 2

Examine pre- to post-PA program differences for changes in Specific Aim 1’s outcomes between cancer survivors with and without T2DM. We hypothesize that greater pre- to post-PA program improvements in these outcomes will be observed for cancer survivors with T2DM.

### Specific Aim 3

Assess pre- to post-PA program differences for changes in Social Cognitive Theory–based (SCT-based) [58,59] constructs, select psychosocial outcomes, HRQoL, and PA between cancer survivors with and without T2DM. We hypothesize that cancer survivors with and without T2DM will demonstrate pre- to post-PA program improvements in (1**)** SCT-based, PA-related self-efficacy, outcome expectations, enjoyment, social support, and barriers; (2) stress, anxiety, mood, and depressive symptoms, and that cancer survivors with T2DM will have greater improvements than cancer survivors without T2DM; (3) in HRQoL due to greater improvements in physical functioning as well as pain intensity/interference, and (4) overall PA participation due to greater increases in light and moderate PA.

### Exploratory Aim

Characterize epigenetic profiles of biologically relevant pathways related to neurocognition, energy metabolism, and proinflammatory processes in cancer survivors with and without T2DM, and whether changes are observed in these profiles pre- to post-PA program. We hypothesize that distinct epigenetic profiles will be evident between groups but that both groups will show pre- to post-PA program changes in these profiles.

## Methods

### Study Design and Recruitment

**Figure 1:**
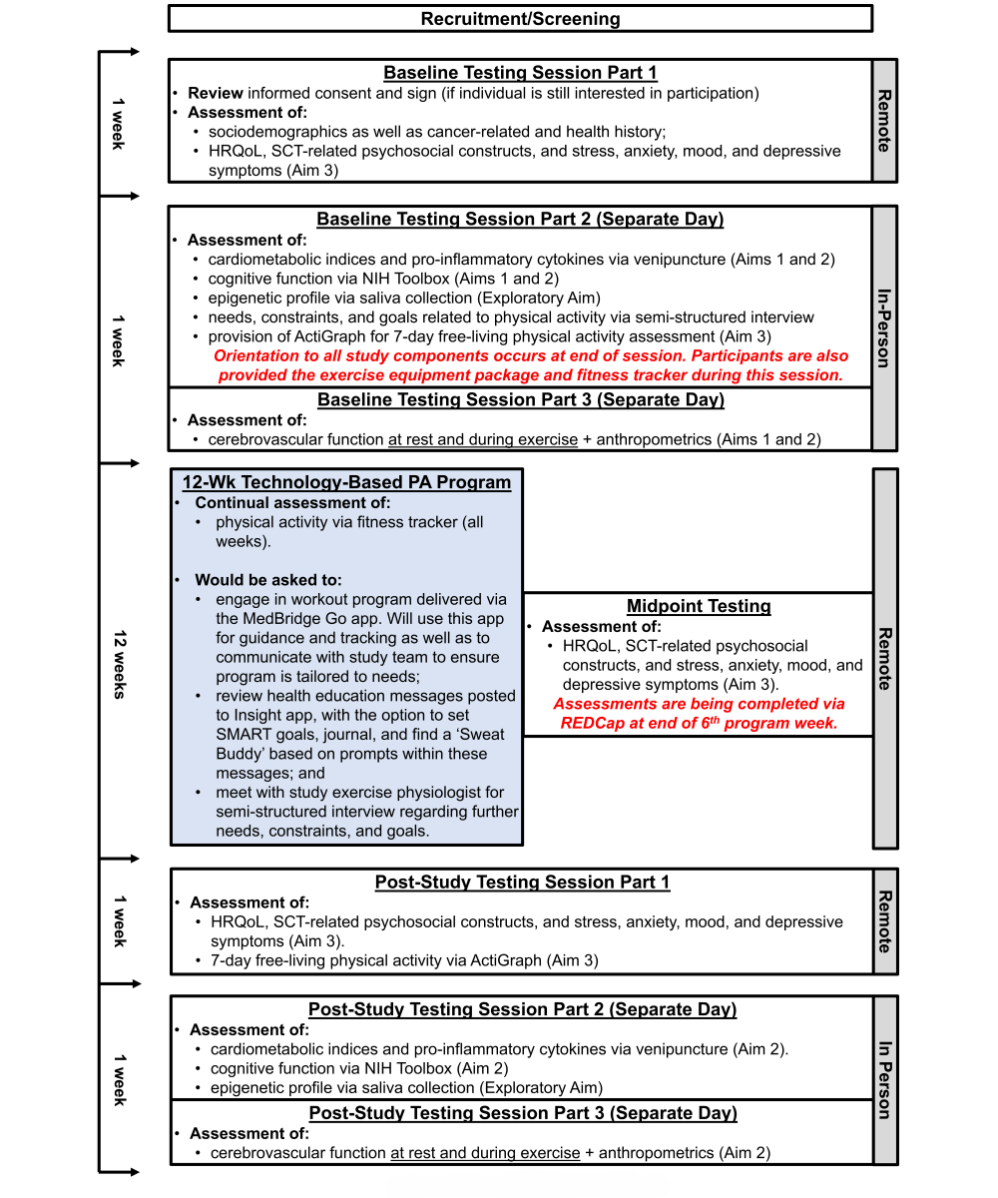
Experimental design. HRQoL: health-related quality of life; NIH: National Institutes of Health; SCT: Social Cognitive Theory.

We are conducting a National Institutes of Health Stage 1 trial [60], and we have registered this trial on ClinicalTrials.gov (NCT06725953). Moreover, we used the SPIRIT (Standard Protocol Items: Recommendations for Interventional Trials)-Outcomes Guidelines to guide the construction of this manuscript [61]. We are recruiting individuals within Oklahoma City and the greater state of Oklahoma. Our accrual target is 38 participants to allow our desired number of 30 participants to be achieved when accounting for an anticipated 20% attrition rate [62]. We are conducting rolling recruitment and are recruiting via (1) emailed and posted flyers; (2) digital advertising to local survivorship groups; (3) oncologist referral; and (4) chart reviews/assistance from the Stephenson Cancer Center Clinical Trials Office. Potentially eligible individuals are sent a REDCap-based (Research Electronic Data Capture–based) screening survey, and, whenever possible, we are verifying cancer diagnosis, chemotherapy regimen, and T2DM status via medical records. We are evaluating individuals against stringent eligibility criteria. Specifically, our inclusion criteria are as follows: (1) 18 years or older; (2) ability to speak/read English; (3) ability to provide informed consent; (4) underwent treatment within the last 3 years for a non–brain-related cancer with said treatment having included chemotherapy and not currently undergoing any active cancer treatments (ongoing endocrine/hormonal therapy or immunotherapy, when either are used for maintenance purposes, are acceptable); (5) self-reported cognitive difficulties following cancer treatment; (6) (for cancer survivors with T2DM) current T2DM diagnosis as classified by a fasting blood glucose of ≥126 mg/dL, 2-hour oral glucose tolerance test of ≥200 mg/dL, HbA_1c_ level of ≥6.5%, or use of medications (eg, Metformin) or insulin to treat hyperglycemia or (for cancer survivors) no current T2DM diagnosis as classified by a fasting blood glucose <100 mg/dL, 2-hour oral glucose tolerance test <140 mg/dL, or HbA_1c_ level of <5.7%. Presence/absence of T2DM to be confirmed preferentially via physician’s documentation or via our measurement of participants’ fasting blood glucose during the blood draws within Part 2 of Baseline Testing (Figure 1); (7) own smartphone or computer with internet access; and (8) willing to participate in the 12-week remotely-delivered PA program. Our exclusion criteria are as follows: (1) reporting a Physical Activity Readiness Questionnaire score indicating PA may be unsafe unless a doctor’s note clearing a prospective participant for study participation can be provided; (2) engaging in ≥75 min/week of vigorous-intensity PA, ≥150 min/week of moderate-intensity PA, or an equivalent combination of both over the last 3 months; (3) currently undergoing active cancer treatment or planning more treatment within the next 3 months; (4) classified within the prediabetes range for their fasting blood glucose measurement (100-125 mg/dL) during Part 2 of Baseline Testing. Notably, those with T2DM verified via a physician’s documentation and who report the use of medications or insulin to treat hyperglycemia, who are found to be in the prediabetes range, will still be enrolled within the T2DM group and study. These results would indicate a participant with well-controlled T2DM, not prediabetes; and (5) currently a prisoner, pregnant, or planning to become pregnant during the study.

We will terminate a participant’s participation if (1) the participant desires to voluntarily withdraw from the study or (2) the investigators feel a participant is unable to complete the study protocol due to changes in health status. All adverse events are being tracked within a REDCap tool that meets Common Terminology Criteria for Adverse Events 5.0 requirements. Adverse events are reviewed by the study’s lead investigators (ZCP, MK, and PM) and the study’s oncologist (CH) and reported to the institutional review board (IRB) as necessary (full protocol available upon reasonable request). We do not currently have a Data and Safety Monitoring Board for this study.

### Study Design

#### Overview

Figure 1 overviews our study design. While participants come to 2 separate laboratories during Baseline and Poststudy Testing Sessions Parts 2 and 3, given the nature of the assessments to be performed, the study is otherwise entirely remote, as shown on the right side of Figure 1. The left side of Figure 1 shows the approximate duration of each study component. We detail each aspect of the Baseline and Poststudy Testing Sessions noted within Figure 1 in the “Brief Aim-Specific Outcome Assessments” subsection. We detail the “12-Week Technology-based PA Program” noted within Figure 1 immediately below.

#### 12-Week Technology-Based PA Program

**Figure 2:**
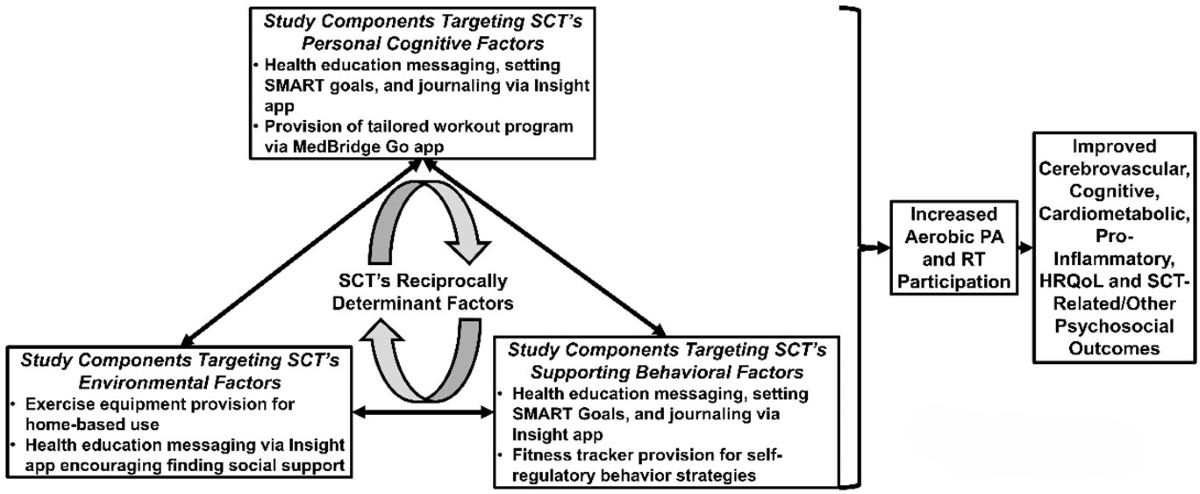
Social Cognitive Theory-informed study components. HRQoL: health-related quality of life; PA: physical activity; RT: resistance training; SCT: Social Cognitive Theory.

We are using a 12-week intervention strategy grounded in the SCT. Figure 2 outlines how this intervention aligns with the SCT’s reciprocally determinant constructs [58,59]: (1) personal cognitive factors; (2) environmental factors; and (3) supporting behavioral factors.

**Figure 3:**
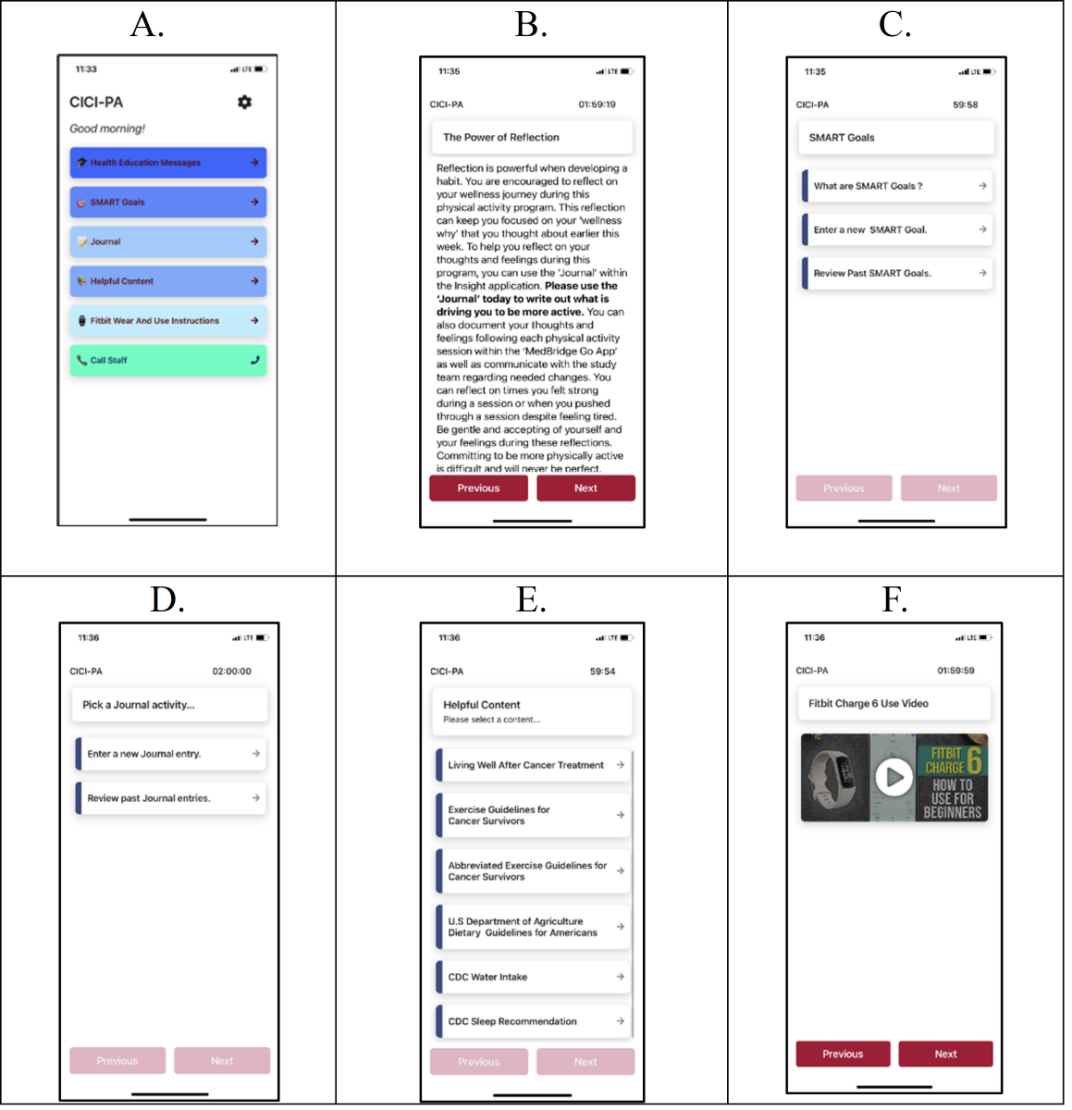
Insight smartphone app. CICI: chemotherapy-induced cognitive impairment; PA: physical activity.

We are using smartphone app (hereafter, “app”) technology to deliver our SCT-informed program. Via an Insight app developed specifically for this study (Figures 3A-3F), participants are receiving health-related educational messaging twice weekly in addition to continued access to information on the benefits of aerobic PA and RT for cancer survivors [63-65]. The health education messages are modeled after our prior studies [66-69]. These health education messages periodically request certain actions, such as setting “SMART Goals” and journaling, with the Insight app having locations where participants can continuously set and review their goals (Figure 3C) and journal entries (Figure 3D). These Insight app-delivered study components are targeting the SCT’s personal cognitive, environmental, and supporting behavioral factors. We developed this study-specific app as a research team and in collaboration with the Stephenson Cancer Center mHealth Shared Resource, which manages the Insight mHealth Platform [70]. While commercially available PA-oriented smartphone apps exist with some of these features, the content we are delivering to participants (enumerated above), as well as the delivery schedule for this content, is unique to our PA program—necessitating a customized app.

**Figure 4:**
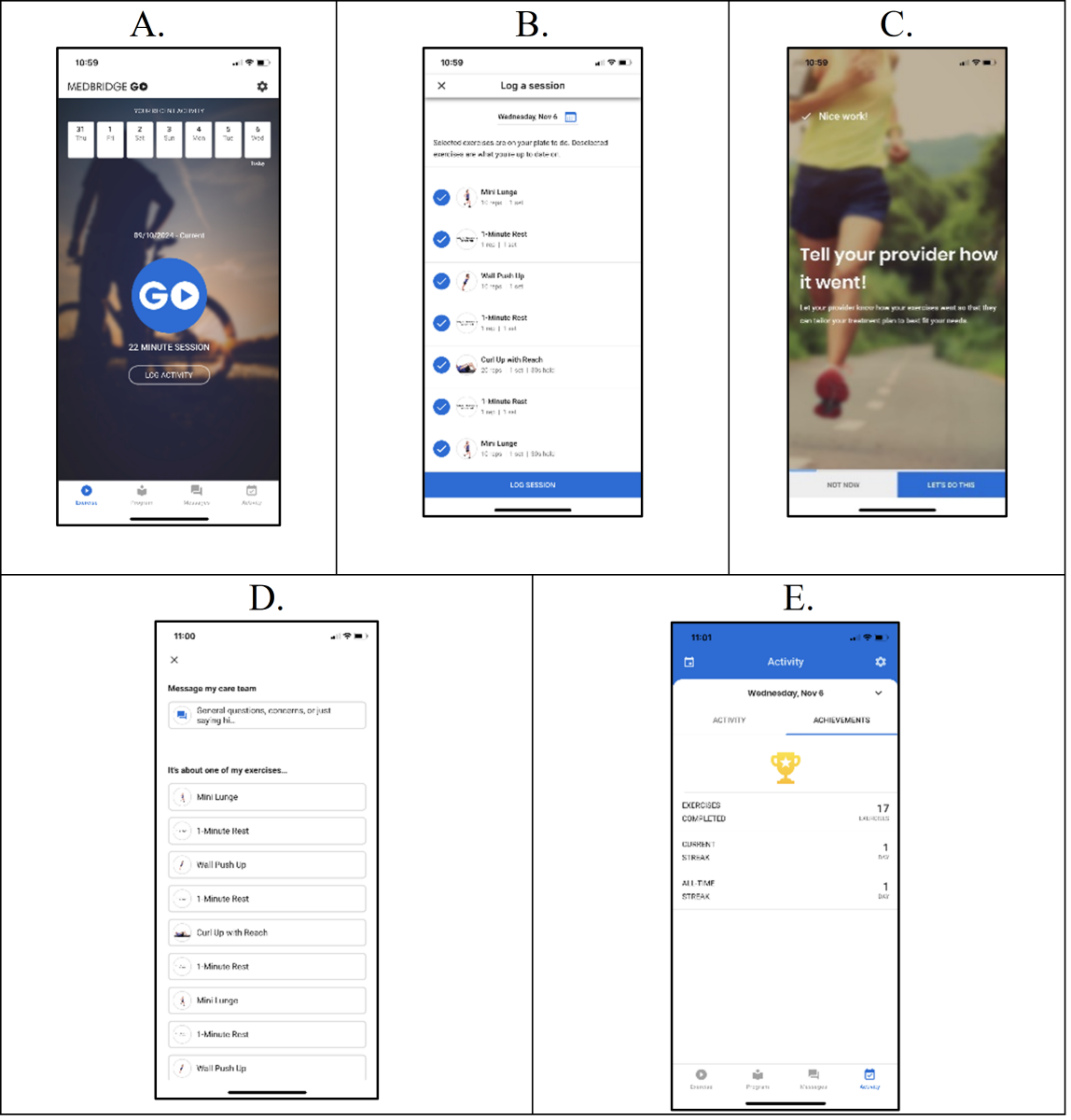
MedBridge GO smartphone app.

The MedBridge GO app (MedBridge Inc) (Figure 4A-E) is being used to deliver workout programs comprised of aerobic PA and RT to participants. We are implementing these workout programs via MedBridge GO, given the MedBridge GO app’s strong security measures and large exercise library (8000+ exercises) in addition to the platform’s ability to measure other items critical to the safe delivery of a PA program (eg, pain and difficulty scales for each prescribed exercise). Participants use MedBridge GO to select days each week on which they desire to complete workouts and set associated reminders (we recommend at least 2 workout sessions/week to all participants). Throughout Baseline Testing, we are collecting information about participants’ access to workout equipment, as well as their needs, constraints, and goals related to PA, via the study’s screening survey and a semistructured interview completed with the study’s exercise physiologist (ZCP). All workouts delivered via MedBridge GO have instructions and associated videos to ensure proper form/safety, and participants use MedBridge GO to note if an exercise was too difficult or if they did not like the exercise. They can also communicate any further needs with the study’s exercise physiologist using MedBridge GO’s messaging feature. Thus, workouts are continuously tailored to each participant throughout study participation based on these different routes of communication. This communication engenders an element of supportive accountability [71] into this SCT-based, technology-delivered PA program. Briefly, the semistructured interviews regarding values and PA-related needs/constraints at Baseline and Midpoint (below), as well as regular communication with the study team via the MedBridge GO app, allow participants to develop a trusting, positive relationship with experts who prioritize their well-being [71]. To further increase the robustness of the PA program delivered via MedBridge GO, we are (1) providing resistance bands to each participant for incorporation into their workouts and (2) conducting another semi-structured interview at study midpoint—adjusting their PA programming for the second 6 weeks of the study in a manner that best meets each participant’s needs. The MedBridge GO workouts/training tutorials and exercise equipment package are targeting the SCT’s personal cognitive and environmental factors. Finally, participants are tracking their PA with the Fitbit Charge 6 during the study, provided during Baseline Testing Session Part 2, and complementing all other intervention components while facilitating self-regulatory behavioral skills. Improving skills, such as goal setting, related to the practice of a behavior, are notable SCT behavioral factors. We connect the Fitbit Charge 6 with Fitabase, a cloud-based platform for obtaining Fitbit/Garmin data [72].

#### Brief Aim-Specific Outcomes and Measurement

##### Sociodemographic and Health Characteristics

We are assessing sociodemographics (age, sex, race/ethnicity, etc) and cancer-related and general health history using our Screening and Baseline Surveys as well as chart reviews before/during Baseline Testing Session Part 1.

##### Cerebrovascular and Cognitive Function as Well as Cardiometabolic, Proinflammatory, and Epigenetic Outcomes

Part 2 of Baseline and Poststudy Testing involves participants coming to one of the study laboratories (Oklahoma City, Oklahoma) following an 8-hour fast, 12 hours of caffeine abstinence, and 24 hours of no alcohol, vitamins, other health supplements, or nonsteroidal anti-inflammatory drug use. They may continue taking medications aligned with health conditions. Participants are also required not to have exercised intensely in the last 24 hours. During this session, participants first complete blood pressure measurements and then blood and saliva sample collections. Blood samples are being collected through traditional venipuncture of the antecubital vein by trained study personnel. Samples are immediately processed and stored at –80 °C. From these blood samples, we assess high- and low-density lipoprotein and total cholesterol, and fasting blood glucose, among other outcomes, using the Lipid Panel+ kit of Piccolo Xpress (Abbott, Chicago, Illinois). Further, we will use enzyme-linked immunosorbent assays to assess high-sensitivity C-reactive protein as well as interleukin-6 and monocyte chemoattractant protein 1—analyses to be run nearer the end of the study to ensure minimal batch effects. These data will allow us to measure cardiometabolic and proinflammatory cytokines aligned with CICI and T2DM pathophysiology [10,11,21]. Finally, we collect saliva with a DNA Genotek OMNIgene Saliva DNA and RNA collection kit (OMR-610; Ottawa, Ontario). Participants spit 1 mL of saliva into the collection tube to a marked fill line, snap the tube closed, and provide it to research staff, after which it is stored at –80 °C. For the purposes of this study, genomic DNA isolations will be performed from whole blood and saliva for epigenetic analyses, namely, DNA methylation. Similar to the enzyme-linked immunosorbent assays, epigenetic analyses will also take place nearer to the end of the study in order to randomize samples to minimize batch effects.

We use the National Institutes of Health Toolbox [73,74] to deploy a standardized cognitive battery following blood and saliva collection and after participants have had the opportunity to have a snack of their choice. This testing includes the following five tests: (1) Dimensional Change Card Sort (executive function); (2) Pattern Comparison Processing Speed; (3) List Sorting Working Memory; (4) Flanker Inhibitory Control and Attention; and (5) Picture Sequencing Memory. These tests will be analyzed per established standards [75,76] to yield a Fluid Composite Score—standards used validly in similar studies examining how exercise impacts the cognitive function of middle-aged and older adults with mild cognitive impairment [77].

We are completing cerebrovascular function and select cardiometabolic outcome measurements during Part 3 of Baseline and Poststudy Testing within a second study laboratory (Norman, Oklahoma). Participants come to this Testing Session following the same fasting and “no exercise” requirements as Part 2 of Baseline and Poststudy Testing (above). We first measure height, weight, and waist and hip circumference, and complete a pregnancy test, if needed. Participants then complete transcranial doppler (TCD) measurements using 2MHz pulse-wave probes and a headpiece (Nuerovision Transcranial Doppler Ultrasound, Multigon Industries, Elmsford, New York). We then have participants complete a test of cerebrovascular reactivity to CO_2_. This test involves participants breathing through a facemask connected to a 3-way sliding valve connected to a meteorological balloon filled with a safe medical-grade gas mixture (CO_2_= 3%, O_2_= 40%, N_2_= 57%) until their partial pressure of end tidal CO_2_ reaches 10 mmHg above baseline levels (takes ≈2 minutes). We complete 2 trials separated by 5 minutes of rest, averaging data across trials per established standards [78,79]. Then, participants’ dynamic cerebral autoregulation is assessed via the Thigh Cuff Release Challenge technique [80]. Large bilateral blood pressure thigh cuffs are rapidly inflated (rapid cuff inflation/deflation system; D.E. Hokanson, Inc., Bellevue, Washington) to occlude the lower limbs. Blood flow is monitored in the dorsalis pedis artery, and the initial thigh cuff pressure starts at 20 mmHg above systolic blood pressure. If blood flow is still detected, thigh cuff pressure increases gradually until occlusion is confirmed. The occlusion is held for 2 minutes. After 2 minutes of occlusion, the cuff pressure is rapidly deflated [80]. Both of these tests are completed while the participant is in a supine position. A second set of TCD measurements is then made while participants engage in a graded exercise protocol on a recumbent bike (Lode Corival Recumbent CPET; Groningen, The Netherlands) to assess fitness. This graded exercise protocol starts with 3 minutes of baseline rest. Following this rest period, the protocol then begins at 20W and increases by 20W increments every 3 minutes until volitional exhaustion or the inability to maintain at least 50 revolutions per minute on the bike. Participants report their rating of perceived exertion at the end of every 3-minute stage (0=no exertion; 10=maximal exertion).

The above measurements provide the following metrics: middle cerebral artery velocity and cerebrovascular resistance, conductance, and pulsatility indexes. As cerebral blood pressure autoregulation difficulties can result from mechanisms underlying CICI [25], we will also use transfer function analysis of TCD measurements, as well as the autoregulation index, to measure dynamic cerebrovascular autoregulation. Analyses will be performed using the most recent Cerebrovascular Research Network recommendations [81]. Other relevant measurements generated during this testing include (1) heart rate calculated by R-R intervals using a Bluetooth II-lead ECG (EQ Life Monitor, Equivital Limited, Cambridge, United Kingdom); (2) mean arterial blood pressure, cardiac output, stroke volume, and total peripheral resistance measured via finger photoplethysmography (Finapres NOVA, Finapres Medical Systems B.V, Enschede, The Netherlands); (3) prefrontal cortex oxygen saturation assessed using wireless near-infrared spectroscopy using a small probe placed unilaterally on the right-side of the forehead (Portalite, Artinis Medical Systems, Elst, The Netherlands); (4) arterial blood oxygenation evaluated using finger-tip oximetry on the right index finger (Oximeter pod, ADInstruments, Sydney, New South Wales, Australia); and (5) end-tidal CO_2_, partial pressure end-tidal CO_2_, and maximum oxygen consumption collected and analyzed via a gas analyzer and pneumotachometer on a breath-by-breath basis (Gemini End-Tidal O_2_ and CO_2_ Analyzer, CWE Incorporated, Ardmore, Pennsylvania).

##### SCT-related Construct, Psychosocial, HRQoL, and PA Outcomes

We are measuring all Aim 3 outcomes during Part 1 of participants’ Baseline and Poststudy Testing Sessions, as well as during Midpoint Testing. The exception is PA, which is only measured at Baseline and after the 12th study week. We are assessing SCT-related psychosocial outcomes using validated surveys delivered via REDCap. Self-efficacy is being assessed using a 9-item measure from Rodgers et al [82]. Participants are noting their confidence during different PA-related situations (“0%: Not confident at all” to “100%: Extremely confident”; 10% increments). Social support is being evaluated using the Social Support and Exercise Survey, with participants reporting frequency of PA support over the last 3 months from family and, separately, friends (“1: None” to “5: Very often”) [83]. PA-related enjoyment is being measured via the 18-item Physical Activity Enjoyment Scale [84], with feelings related to PA-related statements/situations reported on a 7-point scale (varied response options). We are using a 14-item scale [85] to assess agreement between hypothetical PA barriers and participants’ perceived barriers (“1: Strongly disagree” to “4: Strongly agree”). Last, we are using the 13-item Outcome Expectations for Exercise Scale-2 [86] to assess agreement with listed positive and negative benefits of PA (“1: strongly agree” to “5: strongly disagree”).

We are assessing stress, anxiety, mood, and depressive symptoms and HRQoL using validated instruments. We are measuring chronic stress with the 10-item Cohen Perceived Stress Scale [87]. Participants report stress frequency over the last month (“0: Never” to “4: Very often”). We measure chronic anxiety with the State-Trait Anxiety Inventory [88,89], which includes 20 items, each on state and trait anxiety (“1: Almost never” to “4: Almost always”). Chronic mood is being assessed by the Positive and Negative Affect Scale [90]. This scale has 10 items, each for positive and negative affect (“1: Very slightly or not at all” to “5: Extremely”). Chronic depressive symptoms are being assessed by the 9-item Patient Health Questionnaire-9 [91] (“0: Not at all” to “3: Nearly every day”). For HRQoL, the following components of physical and psychological health are being assessed using the Patient-Reported Outcomes Measurement Information System: physical functioning, fatigue, anxiety, depression, pain interference, pain intensity, sleep impairment, and sleep disturbance [92,93]. Measurements are on a 5-point scale, with 5 being the worst on all components aside from physical function, where higher scores are better.

We are assessing PA between Baseline Testing Session Parts 2 and 3 and just after the 12th study week with ActiGraph wGT3X-BT accelerometers—valid for free-living PA assessments [94]. Established protocols for placement and wear duration are being followed by participants [95], and accepted thresholds (≥10 hours/day of wear time; at least 4 days, including one weekend day) are used to clean these data. Established cut points will be used to analyze PA durations per day [96]. Daily average minutes of moderate-to-vigorous PA, light PA, and sedentary behavior will be reported.

### Statistical Analyses

We will first inspect all data for outliers/errors and normality, with analyses done in SPSS (version 29; IBM Corp) and R (R Foundation), depending on the metrics being analyzed. Baseline differences between cancer survivors with and without T2DM for (1) sociodemographics, (2) health characteristics, and (3) Aim 1/Exploratory outcomes (ie, cerebrovascular and cognitive function, cardiometabolic outcomes, proinflammatory cytokines, epigenome) will be compared using *t* tests or chi-square tests, as appropriate. Repeated measures ANOVA will analyze between-group differences for changes in Aim 1/Exploratory outcomes during the PA program (Aim 2) and Aim 3 outcomes (ie, SCT-related constructs, psychosocial outcomes, HRQoL, PA). If any sociodemographic or health characteristics differ at baseline between cancer survivors with and without T2DM, we will use repeated measures ANCOVA. Consistent with recommendations for pilot research [97,98], no a priori power analyses have been performed. Indeed, this study is designed to generate the data necessary to develop larger, properly powered, and federally funded studies on this critical topic.

### Ethical Considerations

This study was approved by the University of Oklahoma Health Campus Institutional Review Board (IRB #17332). Participants consent to the study prior to engaging in any study activity. Further, participants can opt out of participation at any point during the study period. All participant data is deidentified, with data accessible to only those on the study team. Participants are compensated with a US $132 gift card upon completion of all study tasks, covering their time participating and travel, while also being able to keep resistance bands and a Fitbit Charge 6.

### Privacy, Confidentiality, and Safety

All data are being securely stored in REDCap and on encrypted University servers. As this is a Multiple Principal Investigator (MPI) study, the MPIs are jointly responsible for monitoring the safety environment of participants and ensuring that appropriate medical care and coverage are provided to all participants, if necessary. The safety monitor (study oncologist; CH) has also pledged to oversee participant safety (eg, reported adverse events). The MPIs are specifically responsible for monitoring all procedures during the conduct of the study for each participant, including eligibility, enrollment, data collection, evaluation of study outcomes, problems with informed consent, and participant safety and well-being.

## Results

We received funding for this study in July 2024 from the Harold Hamm Foundation via a Harold Hamm Diabetes Center-Stephenson Cancer Center Team Science Grant (Award Number: HR23-061-2). Initial IRB approval was received in July 2024 from the University of Oklahoma Health Sciences, with the most recent IRB modification approval received on 09/21/2025 (IRB #: 17332). We started recruiting cancer survivors in March 2025, and we expect to recruit participants until late 2026. We will begin analyzing baseline data in early 2026.

## Discussion

### Principal Observations

We are investigating (1) whether cancer survivors with T2DM have differential cerebrovascular, cognitive, proinflammatory, cardiometabolic, and epigenetic profiles versus cancer survivors without T2DM and (2) if a 12-week SCT-based [58,59], technology-delivered PA program results in differential between-group changes in these outcomes as well as SCT-related constructs, select psychosocial metrics (stress, anxiety, mood, etc), HRQoL, and PA. Compared to cancer survivors without T2DM, we expect cancer survivors with T2DM to have poorer measures of cerebrovascular, cognitive, proinflammatory, and cardiometabolic outcomes, as well as distinct epigenetic profiles, during Baseline Testing. However, we expect cancer survivors with T2DM to demonstrate greater improvements in these outcomes during the 12-week PA program relative to cancer survivors without T2DM. Finally, we expect that both groups will demonstrate similar improvements in SCT-related constructs and select psychosocial metrics pre- to post-PA program, but that, compared to cancer survivors without T2DM, cancer survivors with T2DM will have greater improvements in HRQoL and PA.

### Strengths and Limitations

This study has several strengths. First, we are using an expansive methodology (eg, TCD, venipuncture, saliva collection) and a team science-informed approach to address an important mechanistic question applicable to a high proportion of cancer survivors who have T2DM, or may be at risk for T2DM [26,99], and are currently experiencing cognitive difficulties following cancer treatment. Second, our use of behavior change theory to inform the components and implementation of a PA program personalized to each participant has merit and should help increase the likelihood of the program’s success. Third, and relatedly, the use of technology (2 smartphone apps, a wearable device) to deliver the PA program is notable, as study observations would have implications for how to best provide supportive care to cancer survivors who do not have readily available access to supportive care posttreatment (eg, cancer survivors living in nonurban areas).

Despite the study’s strengths, a few limitations must be considered. First, TCD cannot image the middle cerebral artery diameter; thus, we are holding the middle cerebral artery diameter constant in this study’s analyses. This reduces the ability to measure absolute middle cerebral artery blood flow velocity. However, our test of cerebrovascular reactivity to CO_2_ has a strong relationship (*r*=0.85) with changes in cerebral blood flow [100] and, therefore, helps to partially overcome this limitation. Second, we are recruiting a relatively small sample with limited power to detect significant differences between groups, and, relatedly, there is an absence of a true control group, which may limit the ability to discern true intervention effectiveness. However, the a priori purpose of this study was to gather the data necessary for a future, larger, and fully-powered randomized trial—a trial that would be able to concurrently address both of these limitations. Finally, we are currently recruiting individuals diagnosed with a broad range of cancer types who were treated with chemotherapy. This strategy aligns with the stated study aims and enhances the feasibility of completing this study in a timely manner. Yet, we acknowledge the need to consider specific cancer types that may most benefit from this type of program and, further, how other treatments (eg, radiation) may also be contributing to cognitive difficulties among cancer survivors.

### Future Implications and Plans

We expect the observations made in our study, particularly during the PA program underpinning Aims 2 and 3, to elucidate unique insights regarding the cognitive benefits of PA at the mechanistic level among cancer survivors, particularly those with T2DM. We want to expand this program to include not only a multisite investigation of how a well-defined PA program in cancer survivors with T2DM improves the outcomes discussed, but also how integration of intervention components regarding other health behaviors (eg, proper sleep, nutrition) might provide additional benefits. For example, integrating a low-touch sleep component into this multisite investigation would be prudent given that up to 75% of cancer survivors experience sleep difficulties [101], which has deleterious impacts on cognitive function and HRQoL while also increasing daytime fatigue—the latter lessening the likelihood of PA participation.

## Appendix

Multimedia Appendix 1 Peer review comments from the Harold Hamm Diabetes Center-Stephenson Cancer Center Team Science Grant Committee, Harold Hamm Foundation.

## Abbreviations

CICI: chemotherapy-induced cognitive impairment
HRQoL: health-related quality of life
IRB: institutional review board
MPI: Multiple Principal Investigator
PA: physical activity
REDCap: Research Electronic Data Capture
RT: resistance training
SCT: Social Cognitive Theory
SPIRIT: Standard Protocol Items: Recommendations for Interventional Trials
T2DM: type 2 diabetes mellitus
TCD: transcranial doppler

## Data Availability

The datasets generated or analyzed during this study are not publicly available due to the pilot nature of this study, but are available from the corresponding author on reasonable request.
