# Supplementary material for: Smartphone-Based Physical Activity Program to Reduce “Chemo-Brain” Symptoms and Improve Health in Cancer Survivors With and Without Type 2 Diabetes: Protocol for a Single-Arm Pre-Post Pilot Trial
Source: JMIR Res Protoc. 2025 Dec 10;14:e79739. doi: 10.2196/79739 (PMC12739459; doi:10.2196/79739)
Supplement: Multimedia Appendix 1 [file resprot_v14i1e79739_app1.pdf]

Harold Hamm
Diabetes Center

May 24, 2024

Zachary Pope, PhD, ACSM-EP
Assistant Professor
Department of Promotional Sciences
OUHSC Hudson College of Public Health

Re: HHD-SCC Team Science Project – Notice of Award

Dear Dr. Pope,

Congratulations! I am pleased to inform you that your grant proposal titled "**Cerebrovascular, Cognitive, Pro-Inflammatory, and Cardiometabolic Outcomes in Cancer Survivors with and without Type II Diabetes Reporting Chemo-Brain: Baseline Differences and Impact of Activity**" will be funded by the Harold Hamm Foundation in the amount of $100,000.

Please see the attached grant proposal feedback from our external, internal and committee reviewers. While there was high enthusiasm for your project, even the proposals that were favorably scored had suggestions for further improvement. We encourage you to address any critiques and modify your project accordingly, to further enhance the quality of your research.

Please work with your Office of Research Administration Sponsored Programs Administrator to start the process of setting up your grant with Grants & Contracts Accounting. The funding period is 12 months beginning July 1, 2024 and ending June 30, 2025. Please consider this letter the Notice of Award (NOA) of the above-reference funds. These funds are subject to the award terms and conditions listed in the solicitation.

A progress report is due to HHDC within 30 days of project termination. Timely submission of the report is a requirement for this award. It is the responsibility of the PI to submit the Progress Report. HHDC will provide a Progress Report template with detailed descriptions for each section to the PI. Of note, the report shall include a summary of: 1) research results, 2) significant discoveries, outcomes and progress in layman's terms, 3) extramural funding applications submitted, resubmitted or planned, 4) funding that has been secured with the help of HHDC and SCC dollars, 5) manuscripts published, submitted, or in preparation, 6) presentations at professional meetings, and 7) any technology development and patents derived from HHDC and SCC funds. Renewed program funding is contingent on HHDC approval of a competitive renewal application.

Please make sure to acknowledge funding from the HHDC and SCC on any publications resulting from this work.

We look forward to the advances in your work that will be made possible by this award. Thank you for your contributions to the field and your involvement with HHDC and SCC.

Sincerely,

Jacob E. (Jed) Friedman, Ph.D.
DIRECTOR, Harold Hamm Diabetes Center
Associate Vice-Provost for Diabetes Programs | Chickasaw Nation Endowed Chair
Professor of Physiology | Pediatrics, Division of Endocrinology and Metabolism | Biochemistry & Molecular Biology
The University of Oklahoma Health Sciences Center, School of Medicine

1000 N. Lincoln Blvd., Ste. 1200, Oklahoma City, OK 73104-3252 | P: 405.271.2824 | F:405.271.2836
HAMMDIABETESCENTER.ORG

Harold Hamm Diabetes Center
Novel Pilot Summary of Review

Principal Investigator: Zachary Pope, PhD

Funding Mechanism: Team Science

Reviewer Overall Scores:

External 1: 2 External 2: 5 Internal 1: 1 Committee: 2

Summary Statement:

This is an application requesting pilot funding to collect essential preliminary data to test the hypothesis that physical activity can impact adverse cognitive effects following chemotherapy in cancer survivors without or with type 2 diabetes. The problem is significant and important to understand. There is a detailed description for patient recruitment, including inclusion and exclusion criteria. The physical activity protocol is also very well described. Several appropriate outcome measurements will be measured including indices of cerebrovascular function, objective measures of cognitive function, and cardiometabolic indices. The weakness pointed out by one reviewer is that this experimental design does not have a no-physical-activity control group. To counter that argument, it is important to remember that this is pilot funding to determine if there is a change in parameters following the physical activity intervention. While a larger study would include a non-activity control, for this purpose, the goal is to see if there is a change within an individual. Essentially, each subject is serving as their own control.

Overall Score: 2

Please use the following NIH Grant scoring criteria when assigning scores, from 1 (highest) to 9 (lowest). Start with a score of 5 and go up or down accordingly. Please use the complete scoring range:

**Degree of Impact**	**Impact Score**	**Descriptor**	**Additional Guidance on Strengths/ Weaknesses**
High	1	Exceptional	Exceptionally strong with essentially no weaknesses
	2	Outstanding	Extremely strong with negligible weaknesses
	3	Excellent	Very strong with only minor weaknesses
Moderate	4	Very Good	Strong but with numerous minor weaknesses
	5	Good	Strong but with at least one moderate weakness
	6	Satisfactory	Some strengths but also some moderate weaknesses
Low	7	Fair	Some strengths but with at least one major weakness
	8	Marginal	A few strengths and a few major weaknesses
	9	Poor	Very few strengths and numerous major weaknesses
**Definitions:**<br>**Minor**: easily addressable weakness that does not substantially lessen the impact of the project. **Moderate**: weakness that lessens the impact of the project.<br>**Major**: weakness that severely limits the impact of the project.			

Harold Hamm Diabetes Center

May 29, 2025

Zachary Clark Pope, PhD, ACSM-EP
Assistant Professor
TSET Health Promotion Research Center, Stephenson Cancer Center
University of Oklahoma Health Sciences Center

Re: HHDC-SCC Team Science – Notice of Award Amended

Dear Dr. Pope:

Congratulations! I am pleased to inform you that your grant proposal titled, "**Cerebrovascular, Cognitive, Pro-Inflammatory, and Cardiometabolic Outcomes in Cancer Survivors with and without Type II Diabetes Reporting Chemo-Brain: Baseline Differences and Impact of Activity**" will be funded by the Harold Hamm Foundation in the amount of **$100,000**.

Please work with your Office of Research Administration Sponsored Programs Administrator to start the process of setting up your grant with Grants & Contracts Accounting. The funding period is 12 months beginning July 1, 2025, and ending June 30, 2026. Please consider this letter the Notice of Award (NOA) of the above-reference funds. These funds are subject to the award terms and conditions listed in the solicitation.

A final report is due to HHDC within 30 days of project termination. Timely submission of the report is a requirement for this award. Team Science projects are eligible for up to three years of funding. Applications for second and third years of funding will be considered through a competitive process and are contingent on the availability of funds, satisfactory progress, and compliance with the program guidelines. Additional guidance on the timeline for renewal applications will be shared in the Fall of 2025.

We look forward to the advances in your work that will be made possible by this award. Thank you for your contributions to the field and your involvement with HHDC.

Sincerely,

Jacob E. Friedman

Jacob E. (Jed) Friedman, Ph.D.
DIRECTOR, Harold Hamm Diabetes Center
Associate Vice-Provost for Diabetes Programs | Chickasaw Nation Endowed Chair
Professor of Physiology | Pediatrics, Division of Endocrinology and Metabolism | Biochemistry & Molecular Biology
The University of Oklahoma Health Sciences Center, School of Medicine

HammDiabetesCenter.org

Administration | 1000 N. Lincoln Blvd., Suite 1200

Oklahoma City, OK 73104 P: (405) 271-2824 | F: (405) 271-2836
